# 

**Published:** 1848

**Authors:** 


WHOLE SERIES—VOL. V, NO. III.

VOL. I.] AUGUST AND SEPTEMBER. [No. 3.

### THE NORTH-WESTERN MEDICAL AND SURGICAL JOURNAL.

PUBLISHED EVERY OTHER MONTH.

EDITED BY

WILLIAM B. HERRICK, M.D, AND JOHN EVANS, M.D.,

PROFESSORS IN RUSH MEDICAL COLLEGE, CHICAGO.

AT TWO DOLLARS PER ANNUM IN ADVANCE.

CHICAGO AND INDIANAPOLIS:

PUBLISHED BY JOHN W. DUZAN.

WM. ELLIS, PRINTER.

1848.

ENLARGED SERIES—VOL. III, NO. III.

